# Influences of gender on cardiovascular disease risk factors in adolescents with and without type 1 diabetes

**DOI:** 10.1186/s13633-016-0026-6

**Published:** 2016-04-19

**Authors:** Talia L. Brown, David M. Maahs, Franziska K. Bishop, Janet K. Snell-Bergeon, R. Paul Wadwa

**Affiliations:** Colorado School of Public Health, 13001 East 17th Place, Aurora, CO 80045 USA; Barbara Davis Center for Childhood Diabetes, University of Colorado Denver, 1775 Aurora Court, Mail Stop A140, Aurora, CO 80045 USA

**Keywords:** Type 1 diabetes, Cardiovascular, Adolescent, Gender differences

## Abstract

**Background:**

Women with type 1 diabetes (T1D) have a four-fold increased risk for cardiovascular disease (CVD) compared to non-diabetic (non-DM) women, as opposed to double the risk in T1D men compared to non-DM men. It is unclear how early in life CVD risk differences begin in T1D females. Therefore, our objective was to compare CVD risk factors in adolescents with and without T1D to determine the effects of gender on CVD risk factors.

**Methods:**

The study included 300 subjects with T1D (age 15.4±2.1 years, 50 % male, 80 % non-Hispanic White (NHW), glycated hemoglobin (A1c) 8.9±1.6 %, diabetes duration 8.8±3.0 years, BMI Z-score 0.62±0.77) and 100non-DM controls (age 15.4±2.1 years, 47 % male, 69 % NHW, BMI Z-score 0.29±1.04). CVD risk factors were compared by diabetes status and gender. Multivariate linear regression analyses were used to determine if relationships between diabetes status and CVD risk factors differed by gender independent of differences in A1c and BMI.

**Results:**

Differences in CVD risk factors between T1D subjects and non-DM controls were more pronounced in girls. Compared to boys with T1D and non-DM girls, T1D girls had higher A1c (9.0 % vs. 8.6 % and 5.1 %, respectively), BMI Z-score (0.70 vs. 0.47 and 0.27), LDL-c (95 vs. 82 and 81 mg/dL), total cholesterol (171 vs. 153 and 150 mg/dL), DBP (68 vs. 67 and 63 mmHg), and hs-CRP (1.15 vs. 0.57 and 0.54 mg/dL) after adjusting for Tanner stage, smoking status, and race/ethnicity (*p* <0.05 for all). In T1D girls, differences in lipids, DBP, and hs-CRP persisted even after adjusting for centered A1c and BMI Z-score.

Testing interactions between gender and T1D with CVD risk factors indicated that differences were greater between girls with T1D and non-DM compared to differences between boys with T1D and non-DM. Overall, observed increases in CVD risk factors in T1D girls remained after further adjustment for centered A1c or BMI Z-score.

**Conclusions:**

Interventions targeting CVD risk factors in addition to lowering HbA1c and maintaining healthy BMI are needed for youth with T1D. The increased CVD risk factors seen in adolescent girls with T1D in particular argues for earlier intervention to prevent later increased risk of CVD in women with T1D.

## Background

Although microvascular outcomes for people with type 1 diabetes (T1D) have improved, cardiovascular disease (CVD) remains the leading cause of death for people with T1D [1] and mortality is still higher than in adults without diabetes (non-DM) [2]. Atherosclerotic changes are known to begin in adolescence in the general population [3–5] and CVD occurs earlier in life and more frequently among people with T1D than in the general population [6]. Women with T1D have at least a four-fold increased risk of CVD compared to non-DM women, as opposed to a doubling of risk in men with T1D compared to non-DM men [7–9]. It is not clear how early in life CVD risk differences begin in females with T1D.

Several other factors have been identified as contributing to increased CVD risk in adults with T1D compared to non-DM including glycemic control and adiposity. Intensive insulin therapy resulted in lower glycated hemoglobin (A1c), reduced CVD events and mortality in the Diabetes Control and Complications Trial (DCCT)/Epidemiology of Diabetes Interventions and Complications (EDIC) study [10, 11]. However, in the DCCT more intensive glycemic control was associated with increased BMI, which was more pronounced in adolescents than in adults [12]. Similarly in adolescents, data from the SEARCH for Diabetes in Youth (SEARCH) study indicate that adolescents with T1D are frequently overweight and obese (22.1 % and 12.6 %, respectively) and were more likely to be overweight than non-diabetic children assessed by the National Health and Nutrition Survey (NHANES) [13]. While not explored in this study, more intensive insulin treatment is likely a contributor [13]. In a recent multinational comparison from the T1D Exchange and the DPV registry median BMI values in youth with T1D were greater than international and their respective national reference values [14].Thus, the added improvement of one major CVD risk factor (A1c) can lead to worsening of another (BMI). However, both weight and glycemic control can be especially difficult for girls with T1D, though it is unclear if increased BMI and A1c for girls during adolescence are the sole factors contributing to increased CVD risk, or if there are other possible risks in girls that are currently unaccounted for [15].

Therefore, our objective was to compare CVD risk factors – glycemic control, BMI, cholesterol, blood pressure, and inflammation - in girls compared to boys to determine how gender affects the relationship of diabetes status with CVD risk. Next, we sought to examine whether these differences are independent of differences in glycemic control and weight by gender and diabetes status.

## Methods

### Study subjects

A cross-sectional study design was used to assess CVD risk factors in youth with and without T1D. At enrollment, subjects were ages 12-19 years and those with T1D were diagnosed by a pediatric endocrinologist, treated with insulin, had diabetes duration over 5 years at entry into the study, and received care at the Barbara Davis Center for Childhood Diabetes (BDC). Non-diabetic control subjects were friends of study subjects or recruited from campus and community advertisements, and were required to be free from diagnosed diabetes. All had normal fasting glucose and HbA1c levels. No siblings or first-degree relatives of patients with T1D were included. Subjects were excluded for diabetes of any other type, or for a history of abnormal cardiac anatomy or arrhythmia that would preclude the subject from vascular function measurements. The study was approved by the Colorado Multiple Institution Review Board and informed consent and assent (for subjects <18 years of age) was obtained in all subjects prior to participation in the study.

### Study visit

All subjects fasted overnight (≥8 hours) and were asked to refrain from caffeine intake and smoking within 8 hours prior to study visit (to avoid potential effect on vascular measures). Tanner stage for all BDC patients was assessed by a clinician investigator or by the subject’s BDC provider. Non-diabetic subjects were requested to have Tanner stage assessed by a clinician investigator with the option of self-assessment if the subject refused a physical exam. Medical history was obtained with standardized questionnaires including methods of insulin administration (injections vs. insulin pump use), dietary habits, and tobacco use. Blood pressure measurements were obtained after a 5 minute rest using a Dynapulse 5200A (PulseMetric, Inc., San Diego, CA) and 3 measurements were averaged. Height was measured by a wall-mounted stadiometer to the nearest 0.1 cm with shoes removed and weight by a Detecto scale to the nearest 0.1 kg [16, 17]. BMI Z-scores were calculated based on the 2000 Centers for Disease Control and Prevention age and gender specific standards [18].

### Laboratory assays

A1c was measured using the DCA Vantage (Siemens, Malvern, PA) at the Children’s Hospital Colorado (Aurora, CO) main clinical lab. Measurements for total cholesterol, triglycerides, and HDL- cholesterol were performed in the University of Colorado Hospital (UCH) Clinical and Translational Research Center (CTRC) Core lab using Olympus AU400e Chemistry. LDL- cholesterol was calculated using the Friedewald formula [19]. High-sensitivity C-reactive protein (hs-CRP) was measured at the Children’s Hospital Colorado (Aurora, CO) CTRC core lab utilizing a multiplex assay platform Siemens (formally Dade Behring) BNII Nephelometer.

### Statistical analysis

Participant characteristics were stratified by both diabetes status and gender and examined. Data are presented as means ± SD for continuous variables, geometric mean and range for continuous variables with non-normal distribution, or count and % for categorical variables. Triglycerides (Tg) and hs-CRP were log-transformed for analysis due to non-normal distribution and expressed as mean and range in Table 1. Because of multicollinearity between A1c and diabetes status, a centered A1c variable was calculated which represented absolute deviations from the mean A1c in both participants with and without T1D to allow for adjustment of A1c [20, 21]. Use of this statistical method to calculate deviation from the mean within a group allows for comparison of variability from the mean between 2 groups with non-overlapping distributions, which is expected for A1c in populations with and without T1D. Stratified two sample t-tests were used to look at differences across groups for continuous variables, and chi square tests were used to look for differences for categorical variables.

**Table 1:** Unadjusted characteristics by sex and diabetes status

Variable	Non-diabetic controls *N* = 100		Type 1 diabetes subjects *N* = 300	
	Boys (*n* = 47)	Girls (*n* = 53)	Boys (*n* = 152)	Girls (*n* = 148)
Age, years^1^	14.9 ± 2.1	15.8 ± 2.0	15.6 ± 2.1	15.2 ± 2.1
Race-ethnicity, % NHW^3^	66 %	72 %	80 %	80 %
Type 1 diabetes duration, years	--	--	8.9 ± 2.9	8.6 ± 3.1
Current smoker, %^2^	2 %	9 %	11 %	3 %
Tanner stage, %^1,2^				
I	6 %	2 %	5 %	1 %
II	17 %	2 %	12 %	3 %
III	9 %	20 %	10 %	10 %
IV	34 %	21 %	20 %	35 %
V	34 %	55 %	53 %	51 %
A1c, %^2,3,4^	5.3 ± 0.3	5.3 ± 0.3	8.7 ± 1.5	9.2 ± 1.7
Centered A1c^2^	0.0 ± 0.3	0.0 ± 0.3	-0.3 ± 1.5	0.2 ± 1.7
BMI Z-score^2,3,4^	0.18 ± 1.14	0.39 ± 0.94	0.46 ± 0.78	0.76 ± 0.71
Total cholesterol, mg/dl^2,4^	142 ± 27	149 ± 29	148 ± 29	165 ± 36
Triglycerides^a^, mg/dl	70(34-163)	82(43-235)	74(27-326)	78(28-394)
HDL-c, mg/dl^1,2,4^	46 ± 8	50 ± 10	49 ± 9	54 ± 11
LDL-c, mg/dl^2,4^	81 ± 23	81 ± 23	83 ± 22	94 ± 29
SBP, mmHg^1,2,3,4^	111 ± 9	107 ± 8	115 ± 9	112 ± 8
DBP, mmHg^2,3,4^	64 ± 6	64 ± 6	68 ± 7	69 ± 6
Hs-CRP^a^, mg/dl^2,4^	0.31(0.03-5.85)	0.48(0.02-9.07)	0.46(0.05-9.2)	0.91(0.04-22)

^a^geometric mean (range)

NHW = non-Hispanic White

^1^ *p* < 0.05 for boys vs. girls Non-DM

^2^ *p* < 0.05 for boys vs. girls with type 1 diabetes

^3^ *p* < 0.05 for Non-DM vs. type 1 diabetes boys

^4^ *p* < 0.05 for Non-DM vs. type 1 diabetes girls

The relationship of gender and CVD risk factors were compared between youth with T1D and non-DM youth. Multivariable linear regression was used to calculate adjusted least square means to test the relationships of CVD risk factors by gender stratified by diabetes status. Interactions for the relationship between diabetes status and the CVD risk factors by gender were tested. Interactions with p-values less than 0.10 were considered significant. Pair-wise comparisons were made for CVD risk factors stratified by gender and diabetes status. CVD risk factors adjusted for Tanner stage, race/ethnicity, and smoking status were compared by gender for youth with and without T1D and also by diabetes status for each gender.

Secondary analyses were conducted to separately investigate the influence of A1c and BMI Z-score on CVD risk factors comparing youth with T1D to non-DM youth testing for adjusted least square means and interactions stratified by diabetes status and centered A1c and BMI Z-score. Statistical analyses were performed using SAS software, version 9.3 of the SAS System for Windows.

## Results

A total of 400 participants enrolled in the study (T1D *n* = 300, Non-DM *n* = 100). Distributions for age, gender, current smoking status, and Tanner stage were similar between participants with and without T1D (Table 1). Participants with T1D had mean diabetes duration of 8.8 ± 3.0 years and 55 % were on insulin pumps.

### Gender differences

When stratified by diabetes status and gender (Table 1), girls with T1D had higher A1c compared to boys with T1D (*p* = 0.01). Girls with T1D had higher BMI Z-score (*p* = 0.002), total cholesterol (*p* = <0.0001), LDL-c (*p* = <0.0001), SBP (*p* = 0.003), DBP (*p* = 0.04) and hs-CRP (*p* = <0.0001) compared to boys with T1D. Girls with T1D also had higher HDL-c (*p* = <0.0001) than boys with T1D. In the non-DM participants, boys had higher SBP (*p* = 0.008) than girls and HDL-c (*p* = 0.053) was marginally lower, but there were no significant differences in BMI Z-score, total cholesterol, triglycerides, LDL-c, DBP, or hs-CRP.

Comparing CVD risk factors by diabetes status for each gender, girls with T1D had higher A1c (*p* < 0.0001), BMI Z-score (*p* = 0.005), total cholesterol (*p* = 0.002), LDL-c (*p* = 0.001), SBP (*p* = 0.0006), DBP (*p* < 0.0001), and hs-CRP (*p* = 0.002) compared to non-DM girls. Girls with T1D also had higher HDL-c (*p* = 0.03). In contrast, boys with T1D had higher A1c (*p* < 0.0001), BMI Z-score (*p* = 0.04), SBP (*p* = 0.03), and DBP (*p* < 0.0001), but had similar total cholesterol, triglycerides, HDL-c, LDL-c, and hs-CRP compared to non-DM boys.

Overall, 6.8 % of study participants reported that they were current smokers. Girls with T1D were less likely to be smokers than boys with T1D (*p* = 0.02), while the differences in smoking status between girls with and without T1D (*p* = 0.08), boys with and without T1D (*p* = 0.07), and non-DM boys and non-DM girls (*p* = 0.12) were not significantly different. Because the differences in smoking status reached or approached statistical significance in all comparisons, we adjusted for smoking in subsequent analyses.

Age was significantly different between non-DM boys and girls (non-DM boys: 14.9 ± 2.1 years vs. girls 15.8 ± 2.0 years, *p* = 0.04), while Tanner stage distribution was similar for boys with and non-DM (*p* = 0.06) as well as for girls with and non-DM (*p* = 0.18). As expected, the Tanner stage distribution was significantly different for boys compared to similar age girls with T1D (*p* = 0.001), as well as for non-DM participants in that girls had more advanced Tanner stage compared to boys, regardless of diabetes status (Table 1, *p* = 0.01). We adjusted for Tanner stage instead of age in order to account for any difference in pubertal status related to age difference between groups. There were no significant differences by diabetes status and gender with regards to race/ethnicity, except that a higher percentage of boys with T1D were NHW compared to non-diabetic boys (p = 0.04). Boys and girls with T1D also had similar diabetes duration (*p* = 0.35).

**Fig. 1:**
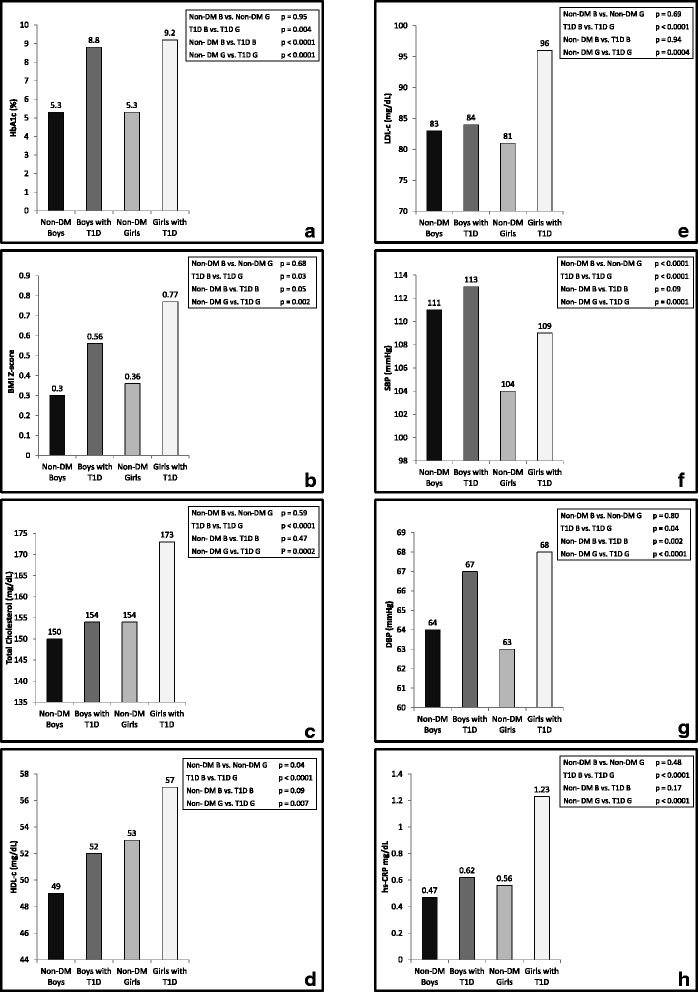
**a**-**h**: Least Square Means for CVD risk factors stratified by gender and diabetes status and adjusted by Tanner stage, race/ethnicity, and smoking status. *P*-values for each respective pair-wise test are also presented in each panel. 1a-HbA1c, 1b-BMI z-score, 1c-total cholesterol, 1d-HDL-c, 1e-LDL-c, 1f-SBP, 1g-DBP, 1h-hs-CRP

Mean levels for CVD risk factors (A1c, BMI Z-score, blood pressure, lipids, hs-CRP) adjusted for Tanner stage, race/ethnicity, and smoking status are shown by diabetes and gender status in Fig. 1. More atherogenic profiles were seen in univariate analysis in girls with T1D compared to both boys with T1D and non-DM girls. Specifically, girls with T1D had higher A1c, BMI Z-score, total cholesterol, LDL-c, DBP, and hs-CRP than boys with T1D and non-DM girls (*p* < 0.05 for all). Additionally, girls with T1D had higher SBP than non-DM girls. Boys with T1D had a similar CVD risk profile compared to non-DM boys, except for higher A1c, BMI Z-score and DBP. Girls with T1D also had higher adjusted HDL-c than boys with T1D and non-DM girls. There were significant diabetes by gender interactions for total cholesterol (p = 0.04), LDL-c (*p* = 0.02), and hs-CRP (*p* = 0.07). These interactions indicate that for these specific CVD risk factors, differences were much greater between girls with and without T1D compared to the differences between boys with and without T1D.

To further investigate the more atherogenic CVD risk profile observed in girls with T1D, secondary analyses were performed with additional adjustment for BMI Z-score and centered A1c, to determine if the interactions by gender and diabetes were independent after accounting for the relationships between CVD risk factors and BMI and A1c (Table 2). Adjustment for centered A1c and BMI Z-score attenuated interactions between diabetes and gender for hs-CRP (*p* = 0.25) and total cholesterol (*p* = 0.12), while gender continued to be a significant effect modifier in the relationship between diabetes status and LDL-c (*p* = 0.04). In spite of the decline of significant interactions, the significant trends in increased least squared means CVD risk factors in girls with T1D that were observed in Table 1 and Fig. 1 remained after further adjustment for centered A1c or BMI Z-score, suggesting that girls with T1D have a worse CVD risk factor profile, independent of their increased BMI Z-score and A1c.

**Table 2:** Cardiovascular disease risk factors by sex and diabetes status adjusted for mean centered A1c and BMI Z-score in addition to race/ethnicity, Tanner stage, and smoking status

Variable	Non-DM		Type 1 diabetes		*P*-value for interaction
Gender	Boys	Girls	Boys	Girls	All
Total cholesterol, mg/dl^(2,4)^	151 ± 6	154 ± 5	156 ± 5	171 ± 5	0.12
Triglycerides^a^, mg/dl	82(70-96)	86(74-99)	83(73-95)	85(73-95)	0.62
HDL-c, mg/dl^(1,2,3,4)^	48 ± 2	53 ± 2	52 ± 2	57 ± 2	0.80
LDL-c, mg/dl ^(2,4)^	84 ± 5	81 ± 4	85 ± 4	94 ± 4	0.04
SBP, mmHg^(1,2,4)^	112 ± 1	105 ± 1	113 ± 1	108 ± 1	0.26
DBP, mmHg^(3,4)^	64 ± 1	63 ± 1	67 ± 1	68 ± 1	0.30
hs-CRP^a^, mg/dl^(2,4)^	0.52(0.34-0.80)	0.62(0.42-0.92)	0.64(0.45-0.91)	1.03(0.72-1.48)	0.25

Data are mean ± SE unless otherwise indicated

^1^ *p* < 0.05 for non-DM boys vs. girls

^2^ *p* < 0.05 for boys vs. girls with T1D

^3^ *p* < 0.05 for Non-DM vs. T1D boys

^4^ *p* < 0.05 for Non-DM vs. T1D girls

^a^denotes geometric least square mean and 95 % confidence interval

### A1c and BMI differences

In a separate analysis, interactions with diabetes status and centered A1c as well as diabetes status and BMI Z-score (data not shown) were examined to assess how the relationship between diabetes status and CVD risk factors varied by A1c and BMI. The same CVD risk factors were considered and the models were adjusted for Tanner stage, race/ethnicity, and smoking status. None of the relationships between diabetes status and CVD risk factors varied by centered A1c, meaning there were no significant interactions. Similarly, the relationship between CVD risk factors and diabetes status did not vary by BMI Z-score, indicating that the relationship between diabetes status and CVD risk profile does not appear to be dependent on the BMI or A1c of the participants.

## Discussion

In this study, adolescent girls with T1D had a significantly worse CVD risk profile compared to non-DM girls and boys with T1D. Specifically, girls with T1D had higher BMI Z-score, A1c, hs-CRP, total cholesterol, and LDL-c. In contrast, adolescent boys with T1D had a similar CVD risk profile to non-DM boys. The higher CRP and lipid levels observed in girls with T1D were not entirely explained by higher BMI and A1c when compared to both boys with T1D and non-DM girls. In addition, secondary analyses indicate that A1c and BMI do not modify the relationship between T1D status and the measured CVD risk factors, while gender appears to modify many of these relationships with more atherogenic CVD risk factors in adolescent girls with T1D. A recent study by Cree-Green et al. similarly found that female, but not male, adolescents with T1D had more atherogenic lipoprotein subfraction distribution which was correlated with insulin resistance [22].

Previous studies indicate that adults with T1D have shorter life expectancies compared to adults without diabetes where women with T1D lose nearly 13 years on average compared to non-diabetic women with 31 % of this difference attributed to heart disease [9, 23, 24]. Other studies have shown that girls with T1D have increased difficulty with maintaining a normal weight and glycemic control during adolescence [15, 25] and have worse lipid levels compared to their male counterparts [26]. The SEARCH for Diabetes in Youth Study found that girls with T1D showed a decrease in health-related quality of life as they aged through adolescence while boys saw an increase in their health-related quality of life during the same ages with physical activity and weight gain being major contributors to sex differences [27]. Other risk factors for increased CVD risk in adolescents with T1D have also been identified, such as increased consumption of sugary drinks [28], poor diet [29] and decreased physical activity [30]. In a previous analysis published on this cohort, we did not observe a difference in diet or physical activity between girls and boys with T1D indicating that these factors are likely not contributing to observed differences within adolescents with T1D [5]. Though, some reports have shown a decrease in physical activity in girls with T1D compared to girls without diabetes [31].

However, the Coronary Artery Calcification in T1D (CACTI) study and other T1D cohorts have shown that adult women with T1D have similar BMI and A1c levels compared to men with T1D [7, 8]. Therefore, it is possible that even when women with T1D are able to improve their weight and glycemic control as adults, damage done during adolescence may still adversely affect their long-term CVD risk. Our findings suggest that women with T1D may have an increased risk for CVD events due to physiologic phenomenon that happens to girls during adolescence, in addition to exposure to higher A1c levels and other CVD risk factors even if these factors are improved in adulthood.

Other potential pathophysiologic pathways that may contribute to elevated early CVD risk in adolescent girls with T1D should be explored. For example, this study could indicate that there is an additional factor or factors that impact both increased CVD risk factors (including BMI) and glycemic control in women with T1D. We know that women and girls with T1D experience increased sex hormone and menstrual dysfunction compared to women and girls without T1D [32] including increased sex-hormone binding globulin and testosterone, as well as decreased estrogen [33, 34]. As a result, females with T1D also have a higher prevalence of polycystic ovarian syndrome [35], late menarche [36], irregular menstrual cycles, amenorrhea, and early menopause [37, 38]. Menstrual dysfunction can persist despite improved glycemic control [39–41]. Therefore, differences in hormones and pubertal processes and interactions with T1D and insulin treatment for the disease could contribute to difficulties achieving optimal glycemic control and weight management, in addition to increased inflammation, with worse effects in girls with T1D. Additionally, the loss of estrogen protection early in adolescence and greater insulin resistance could contribute to the increased dyslipidemia observed in this population [8, 42].

There are limitations to these data. The data for this analysis was collected cross-sectionally and participants with T1D and non-DM may not be representative of the underlying populations from which they were recruited. However, elevated BMI and A1c in adolescent girls with T1D have been observed elsewhere [15, 25]. Our findings expand upon those studies to highlight the increased CVD risk found in adolescent girls with T1D compared to boys with T1D or non-DM adolescent girls. Moreover, the increased CVD risk factors are independent of BMI and A1c.

Future research should focus more on girls with T1D to explore other factors that make this group especially susceptible to increased CVD risk. Additionally, the observed relationships should be investigated over time to understand at what stage in development the weight and glycemic control have more impact on development of CVD in adults with T1D and how gender and CVD risk in adolescence relates to CVD outcomes later in life. Finally, special clinical consideration and therapeutic interventions should be targeted towards young girls with T1D to address CVD risk factors in addition to achieving A1c targets and maintaining healthy weight to prevent later diabetic complications.

## Conclusions

Adolescent girls with T1D have a worse CVD risk profile compared to non-DM girls and boys with T1D. In this study, we found that girls with T1D had higher BMI Z-score, A1c, hs-CRP, total cholesterol, and LDL-c. In contrast, adolescent boys with T1D had a similar CVD risk profile to non-DM boys. Interventions targeting CVD risk factors, lowering HbA1c and maintaining healthy BMI are needed for youth with T1D and may be especially important for adolescent girls with T1D.
